# Research on the Performance of a Liquid–Solid Triboelectric Nanogenerator Prototype Based on Multiphase Liquid

**DOI:** 10.3390/mi16010078

**Published:** 2025-01-11

**Authors:** Wei Wang, Jin Yan, Xianzhang Wang, Hongchen Pang, Chengqi Sun, Yin Sun, Lijun Wang, Dapeng Zhang

**Affiliations:** 1 College of Naval Architecture and Shipping, Guangdong Ocean University, Zhanjiang 524088, China; 2 Guangdong Provincial Key Laboratory of Intelligent Equipment for South China Sea Marine Ranching, Guangdong Ocean University, Zhanjiang 524088, China; 3 College of Mechanical Engineering, Guangdong Ocean University, Zhanjiang 524088, China

**Keywords:** ML-TENG Pro, multiphase-liquid, air–oil–water three-phase, emulsion, power generation

## Abstract

In recent years, liquid–solid triboelectric nanogenerators (L-S TENGs) have been rapidly developed in the field of liquid energy harvesting and self-powered sensing. This is due to a number of advantages inherent in the technology, including the low cost of fabricated materials, structural diversity, high charge-energy conversion efficiency, environmental friendliness, and a wide range of applications. As liquid phase dielectric materials typically used in L-S TENG, a variety of organic and inorganic single-phase liquids, including distilled water, acidic solutions, sodium chloride solutions, acetone, dimethyl sulfoxide, and acetonitrile, as well as paraffinic oils, have been used in experiments. However, it is noteworthy that the function of multiphase liquids as dielectric materials is still understudied. The “Multiphase Liquid Triboelectric Nanogenerator Prototype (ML-TENG Pro)” presented in this paper takes a single-electrode solid–liquid triboelectric nanogenerator as the basic model and uses lubricating oil and deionized water as dielectric materials. After verifying the stability of single-phase liquid materials (e.g., DI water, seawater, ethanol, etc.) for power generation, the power generation performances of oil–water two-phase, gas–oil–water three-phase (with a small number of bubbles), and gas–oil–water three-phase (with many bubbles) in open space are further investigated. COMSOL Multiphysics 6.0 software was used to investigate the material transport mechanism and formation of oil–water two-phase and gas–oil–water three-phase. Finally, this study presents the power generation performance of ML-TENG Pro in the extreme state of gas–oil–water three-phase “emulsification”. This paper outlines the limitations of the ML-TENG, named PRO, and suggests avenues for future improvement. The research presented in this paper provides a theoretical basis for evaluating the quality of lubricants for mechanical power equipment.

## 1. Introduction

As a “transporter” and “collector” of charge energy in nature, the conception of a triboelectric nanogenerator (TENG) was proposed by Wang’s group, based on the principle of contact electrification, in 2012 [1]. This has opened up a new field of exploration for humans to collect and utilize clean energy, such as wind energy and ocean energy. In contrast to traditional thermal power generation (e.g., from coal, oil, and gas) and electromagnetic energy power generation (e.g., from hydropower stations and wind turbines), the triboelectric nanogenerator (TENG) has attracted attention due to its lightweight design and capacity to utilize ’high entropy’ energy. It has also demonstrated clear advantages in decentralized applications. The development of the triboelectric nanogenerator (TENG) in recent decades has resulted in the creation of a vital energy source for use with distributed sensors and wearable devices [2,3,4,5,6]. The fundamental principle of TENG is based on the integration of the following two key effects: contact charging and electrostatic induction. In essence, two distinct dielectric materials are in contact with one another. Friction generates an induced charge, which is transferred from one dielectric material to the other, in accordance with the difference in their electron affinity. This charge is then collected by a wire that transfers it to the outside of the two dielectric materials. There are four principal modes of operation in TENG, as follows: (a) Vertical Contact separation (CS) mode; (b) in-plane Lateral Sliding (LS) mode; (c) Single-Electrode (SE) mode; and (d) Freestanding Triboelectric-Layer (FT) mode [7,8,9,10,11,12,13].

**Figure 1:**
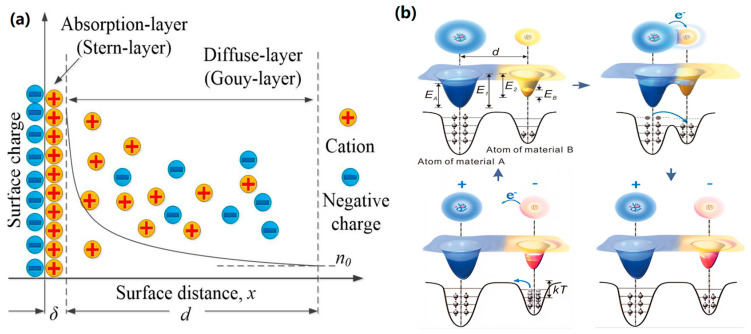
The working mechanism of L-S TENG. (**a**) An illustration of the EDL (electrical double-layer model [32]; (**b**) “Wang transition” model [33].

Furthermore, the low cost of fabrication materials, versatility of structures, reliability of output, high energy conversion efficiency, and environmentally friendly nature of liquid–solid contact electrification have contributed to its growing prominence. This has led to the rapid development of liquid–solid triboelectric nanogenerators (L-S TENGs) in the domains of liquid energy harvesting and self-powered sensing [14,15,16,17,18,19,20,21,22,23,24]. The operational mechanism of the L-S TENG can be elucidated as follows: upon contact between a liquid and a solid surface, the latter generates a triboelectric charge and concurrently establishes an electric double-layer (EDL), as illustrated in Figure 1a, with the objective of neutralizing the charge on the surface of the solid material. When the solid surface is connected to an external electrode and a wire, a charge flow will be generated between the liquid and the solid surface, thereby balancing the potential difference. When the solid surface is connected to an external electrode with a wire, a charge flow is generated between the liquid and the solid surface, which balances the potential difference. However, when it is in contact with water, the CE mechanism at the liquid–solid interface requires further explanation [25]. In 2018, Wang and his research team [26,27,28,29] realized that the occurrence of an electron transfer leads to contact electrification between solid materials, and they advanced an electron cloud overlap model to provide a comprehensive explanation for electron transfer [30], as shown in Figure 1b. In the preliminary stage, materials A and B remain as distinct and separate entities, exhibiting no overlap. The confinement of electrons within the potential well impedes their ability to escape. When interactions occur between the surfaces of two materials, the electron clouds display a notable degree of overlap, thereby transforming the initially symmetrical single-potential well into an asymmetrical double-potential well. This transformation results in a reduction of the potential barrier between the two atoms. Consequently, an electron transfer occurs from one atom to another, initiating contact electrification. Subsequently, upon the separation of the two materials, the transferred electrons persist as fixed charges on the surface of the respective materials. This results in the imparting of positive and negative charges to materials A and B. This theoretical framework provides a detailed account of the conditions under which electron transfer occurs, and it can be designated as the “Wang transition” model. These characteristics of the liquid complicate ion and molecule sorption on solid surfaces, which, in turn, introduces uncertainties regarding the CE mechanism at the liquid–solid interface. Nevertheless, the “Wang transition” model can be extended to encompass contact electrification across this interface. The interaction between liquid and solid surfaces enables interactions between liquid molecules and solid surface atoms, which may result in electron cloud overlap and subsequent electron transfer. In specific instances, atoms that either receive or release electrons from solid surfaces may be transported away from the solid interface by molecules within the liquid, resulting in electron retention on the solid surface and contributing to liquid–solid contact electrification [31].

Given that the liquid triboelectric materials used in L-S TENGs are very easy to obtain in nature, single–phase liquid materials, such as rainwater, tap water, and salt water, are tested for energy generation, as shown in Table 1. On the other hand, many effects caused by contact electrification are unwelcome in our life or industry. Especially for the oil–solid contact electrification generated by low conductivity oil flowing through pipes and filters, for which it is the main cause of many tragedies in the oil industry [33]. Therefore, it is imperative to utilize L-S TENGs in an efficacious manner for the purposes of energy harvesting and application. In contrast to solid–solid TENGs, the triboelectric materials of L-S TENGs comprise liquid material, which serves to further complicate the process of controlling L-S TENGs. There is a pressing need to explore more effective strategies to control liquid–solid contact electrification.

It is noteworthy that, in comparison to continuous and uniform single-phase fluids, multiphase fluids (such as gas–solid, liquid–solid, gas–liquid, and liquid–liquid) still necessitate further investigation as potential triboelectric nanogenerator materials. This is due to the inherent complexities associated with their parameters, phase interface alterations, and the presence of non-equilibrium effects between media. As documented in the referenced reports, Xu, Liu, Zhang, and Wang et al. introduced a Single-Electrode mode WS-TENG, utilizing PTFE as the friction layer, in 2021. The triboelectric charging phenomenon between the sandstorm flow and the surface of the friction layer was then investigated via the sediment–air injection method, wherein the relevant power generation performance parameters of the WS-TENG, as detailed in the article, have potential for use as a self-powered sensor for monitoring sandstorm conveying rates in challenging environments [37]. With regard to the mixing of liquid and solid particles, in 2020, Lei Yang and colleagues introduced a single-electrode triboelectric nanogenerator (PLDD-TENG) driven by the introduction of carrier particle droplets [38]. This was combined with a deep learning method, thereby providing a foundation for guiding the application of real-time monitoring of suspended sediment and sediment in water systems. In a further study, J. Zhao developed an O-S TENG for the real-time monitoring of contaminants such as wear debris, deposited carbon, and organic molecules in lubricating oil, in 2021, under the guidance of ZL Wang [35]. For further development, T. Huang, A. Qin, and colleagues (2023) created an L-S TENG for the detection and classification of microplastic particles in liquids, which was combined with a convolutional neural network deep learning model [39]. In 2022, ZL Wang’s team developed a high-performance TENG (GL-TENG) based on the rheological properties of gas–liquid two-phase flow and a Venturi-like design. It has been demonstrated that the device is capable of achieving ultra-high voltage and volume charge densities, of 3789 volts and 859 millicoulombs/m^3^, respectively. These findings suggest that the technology may offer a promising strategy for energy harvesting and sensing applications [40].

**Figure 2:**
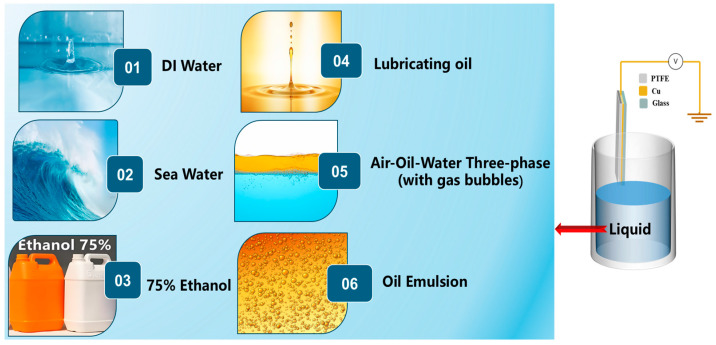
The ML-TENG Pro and its different dielectric materials.

The Multiphase Liquid Triboelectric Nanogenerator Prototype (ML-TENG Pro) described in this paper is based on a typical single-electrode solid–liquid triboelectric nanogenerator, with sea water, lubricating oil, deionized water, and 75% ethanol serving as the dielectric materials [41]. As illustrated in Figure 2, following the verification of the power generation stability of DI water, sea water, 75% ethanol, and oil, the ML-TENG Pro proceeded to examine the power generation performance of phase 1, comprising an oil–water two-phase system; phase 2, comprising three-phase air–oil–water (with a small number of bubbles); and phase 3, comprising air–oil–water (with a large number of bubbles), respectively, in the open space of a round-mouthed cup as a container. COMSOL Multiphysics 6.0 software was used to investigate the material transport mechanism and the formation of oil–water two-phase and air–oil–water three-phase. This paper presents a study of the power generation performance of the ML-TENG Pro in an extreme state of an air–oil–water three-phase ‘emulsion’.

As previously mentioned in the literature review, there is a deficiency of research on “liquid-liquid” multiphase fluids as the materials used for TENG’s power generation. The contributions and advantages of the “ML-TENG Pro” model proposed in this paper are as follows:

Firstly, in contrast to the single-phase fluids examined in prior studies, this study adopts an exploratory approach, investigating the power generation performance of “oil-water” two-phase and “gas-oil-water” three-phase fluids.

Secondly, this study is distinct from previous research on multiphase liquids (e.g., “solid-liquid”, “gas-liquid”, and “contaminated oil”). It focuses on “liquid-liquid” environments used for power generation, especially “oil-water” and “gas-oil-water” liquids. It also assesses the limits of “air-oil-water” liquids, focusing on the electrical energy output of “emulsions”.

Thirdly, this paper uses COMSOL Multiphysics 6.0 software to study the factors involved in the transformation of “oil-water” multiphase fluids to “gas-water-oil”.

The suffix “prototype” signifies that the technology is not ready for practical engineering applications, due to the surface material of ML-TENG being insufficiently oleophobic, resulting in unstable power generation in various fluid types, including oil–water, oil–water–gas, and emulsion. This paper presents a preliminary discussion of this limitation, and provides considerations for future improvements to ML-TENG PRO. Furthermore, considerations for future improvements to ML-TENG PRO are discussed.

## 2. Materials and Methods

### 2.1. Materials, Fabrication, and Experimental Design of ML-TENG Pro

The experiment utilized a 0.5 cm-thick polytetrafluoroethylene (PTFE) film (purchased from Alibaba, PRC e-commerce company, HangZhou, China), a 0.2 cm-thick pure copper film (purchased from Alibaba, PRC e-commerce company, HangZhou, China), a 2 mm-diameter copper wire (purchased from Alibaba, PRC e-commerce company, HangZhou, China), and a quartz glass sheet. The ML-TENG Pro was constructed in a conventional single-electrode configuration. As illustrated in Table 2, the Single-Electrode mode has been the preferred option in a series of recent sensor studies on liquid–solid triboelectric nanogenerators, with a greater frequency of selection than the other types. The rationale behind this is that, in the Single-Electrode mode, a working electrode is linked to an external load, and the other end of the load can be connected to a non-working reference electrode or to the ground, thus facilitating the flow of electrons between the two terminals of the load. This connection method is more conducive to the collection of mechanical energy, and in contrast to other TENG modes, the freely moving electrode enhances the versatility of liquid energy collection. The rationale behind single-electrode power generation was elucidated by Bai et al. in 2022 [42].

(1) $$V_{oc}=\frac{\sigma_{1}}{\varepsilon_{0}}\pi l$$

(2) $$Q_{SC}=\frac{1}{2}\sigma_{f}w$$

whereas, when l goes to infinity and *x*/l, *g*/l are close to zero, $V_{oc}$ and $Q_{SC}$ are expressed by

(3) $$V_{OC}=\frac{\sigma_{t}gx}{\pi \varepsilon_{0}l}ln(l)$$

(4) $$Q_{SC}=\frac{\sigma_{t}wx}{\pi }ln(l)$$

where l and g are the length of the electrode and the gap between the main electrode and reference electrode.

In this configuration, the charge is transferred from the positive friction layer to the negative friction layer, and the output voltage is measured between the positive friction layer, which serves as the electrode, and the other grounded electrode. In this study, the liquid dielectric materials utilized in the positive friction layer are DI water (purchased from a Watsons store, Zhanjiang, China), sea water (outdoor collection), 75% ethanol (purchased from pharmacies), and lubricating oil (the lubricant is Mobil 5W-30 Manufactured by ExxonMobil Co, Avon, TX, USA). As illustrated in Table 1, the dielectric material employed in the negative friction layer is PTFE, a fluorinated polymer with low surface energy, which has garnered significant interest in the field of liquid–solid-coupled TENG, due to its low friction coefficient, hydrophobicity, and exceptional thermal stability.

**Figure 3:**
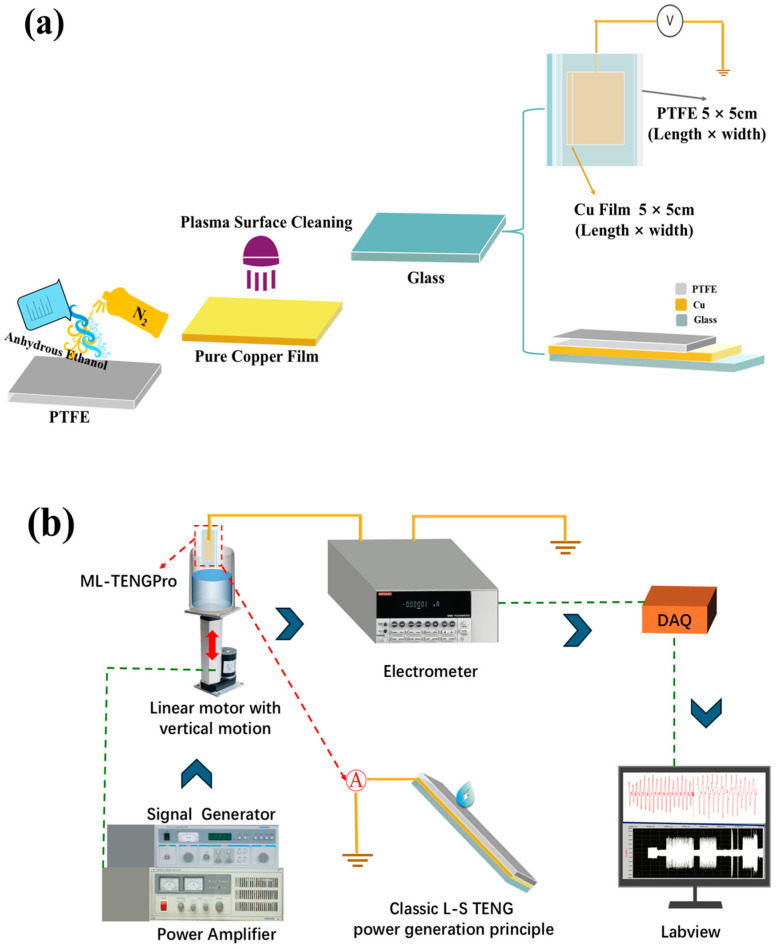
The materials, fabrication, and experimental process of ML-TENGPro. (**a**) gives the manufacturing process for the ML-TENG Pro. (**b**) describes tests of ML-TENG Pro’s power generation performance in various types of liquids.

As illustrated in Figure 3a, the fabrication process of the ML-TENG Pro involved the cutting of a 5 cm × 5 cm PTFE membrane and a quartz glass sheet. To ensure the ML-TENG Pro’s integrity against liquid materials, a 4 cm × 4 cm pure copper film was cut out to prevent contamination of the pure copper conductive electrode by the liquid working medium during the experiment. In particular, prior to assembling the ML-TENG Pro, anhydrous ethanol (purchased from Sigma-Aldrich, St. Louis, MO, USA) was used to repeatedly rinse and clean the PTFE surface for approximately five minutes. Subsequently, high-purity laboratory nitrogen was employed to blow-dry the PTFE surface. Concurrently, a plasma surface cleaning apparatus (FemtoScience, Hwaseong-si, Republic of Korea, 70 W power, 50 Hz frequency, 5 min cleaning time) was employed to clean the pure copper film, thereby ensuring its conductivity. Ultimately, the components were assembled.

As demonstrated in Figure 3b, a variety of devices were employed for the purpose of evaluating the ML-TENG Pro. Initially, a signal generator and power amplifier provided two signals for the vertical movement of the linear motor at high or low frequencies. The positively charged liquid material utilized in the experiment was positioned within an open container, which was then placed on a platform at the terminus of the linear motor. The ML-TENG Pro then comes into constant contact with the liquid dielectric material, as the linear motor moves the rod vertically up and down by opening the response switch when electricity is applied. This results in the charge generated on the PTFE surface being conducted via the copper film and wire attached to the electrometer device. Finally, the electrical signal is recorded via the data acquisition equipment on a computer with LabView software 2022 installed.

### 2.2. Experimental Process for Power Generation Using Water, Lubricating Oil, Two-Phase Water–Oil and Three-Phase Water–Oil–Gas Based on ML-TENG Pro

The experimental setup for the power generation process of the ML-TENG Pro liquid material is presented in Figure 3b. At the beginning of the experiment, a single liquid phase, including DI water, seawater, 75% ethanol, and an engine oil lubricant, was employed as a triboelectric material. Additionally, low-frequency or high-frequency vertical movements were applied to facilitate contact separation between the ML-TENG Pro and the single-phase liquid material, as mentioned above [47]. The objective of this experiment is to demonstrate the efficacy of the ML-TENG Pro, as prepared according to the scheme depicted in Figure 3a. While the surface material PTFE utilized in the ML-TENG Pro may not be fully satisfactory for the oil lubricant’s oil repellency (the experimental data and phenomena related to this aspect will be discussed in further detail later), the power generation effect of the other single liquid phase materials provides clear evidence of the reliability of the ML-TENG Pro preparation process.

The following experiment comprises an oil–water two-phase liquid served as the triboelectric material shown in Figure 3b, with a volume ratio of 3:7 between the oil and water phases. This paper utilizes the “oil-water two-phase liquid” for power generation performance testing experiments. As the frequency of contact and separation between the ML-TENG Pro and the oil–water two-phase liquid increases, the “air-oil-water” three-phase liquid is generated due to the forced motion. The generation mechanism and power generation performance of the phase change of the “air-oil-water” three-phase liquid are documented and discussed in this paper. The generation process of the “air-oil-water” three-phase liquid is briefly described here. As the frequency of relative motion between the ML-TENG Pro and the three-phase working fluid (consisting of oil, water, and gas) increases, the effect on the motion of the three-phase fluid is mainly manifested in two stages. Initially, the fluid exhibits a smooth, stratified flow, which can be described as “wave stratified flow”. Subsequently, intermittent flow phases of “slug flow”, “pseudo-slug flow”, and, ultimately, large-scale emulsification, are observed.

As demonstrated by the research on gas–liquid–liquid three-phase flow, conducted by scholars such as Wu Hao Jiang et al. in 1999 [48], the mentioned “air-oil-water” three-phase fluid is influenced by the following three key factors:

(1) The interaction between the liquid phase and the solid contact wall, specifically the type of liquid phase that make contact with the wall surface. If the oil phase makes contact with the solid wall, the flow is deemed to be oil-based. It is evident that this experiment is not confined to “oil-based flow”, which introduces an additional layer of complexity to multiphase flow.

(2) The proportional relationship between the oil and water phases.

(3) The relationship between the gas phase and the overall “liquid phase”. Based on these considerations, Wu et al. postulated that the oil and water were in a state of mixture and could be regarded as a single liquid phase, with the addition of gas representing a two-phase flow of a “gas and overall liquid phase“. It is evidently the case that Wu et al.’s third hypothesis regarding the “air-oil-water” three-phase liquid significantly simplifies the theoretical analysis. Based on this hypothesis, and in conjunction with Stokes’ law, we present the expression for the bubble velocity distribution under different sizes and Reynolds situations, which are as follows:

When the diameter ($D_{b}$) of the bubble is less than 0.1 mm and 200~300 < Re < 700,

(5) $$v=\sqrt{\frac{4}{3}\cdot \frac{gD_{b}(\rho_{L}-\rho_{G})}{\rho_{L}}}$$

where $\rho_{L}$ and $\rho_{G}$ are the Apparent Density of liquid and gas phases, g gives an acceleration due to gravity.

When the diameter ($D_{b}$) of the bubble is more than 2~3 mm and 700 < Re < 1000,

(6) $$v=\sqrt[{4}]{\frac{4g\sigma (\rho_{L}-\rho_{G})}{{C_{D}}^{2}{\rho_{L}}^{2}}}$$

where $C_{D}$ is the Coefficient of Resistance, σ gives a Surface Tension Coefficient of a bubble.

This paper presents the $C_{D}$ and σ of the bubble, as these parameters are subject to change during their ascent, due to the substantial diameter of the bubble. As the liquid resistance acting on the bubble is combined with the effects of its surface tension, deformation occurs. Furthermore, this deformation affects the trajectory of the bubble.

### 2.3. The ML-TENG Pro’s Ability to Generate Power Under Extreme Conditions—Emulsifying Lubricant

Building on the preceding investigation into the generation of power from an “air-oil-water” liquid triboelectric nanogenerator, this paper utilizes a fully emulsified air–oil–water three-phase liquid dielectric material as the subject of experimental investigations into power generation in the final stage. In accordance with the established experimental conditions, the power generation of the emulsion demonstrated a correlation with the movement of the ML-TENG Pro. However, it is of notable interest that the ML-TENG Pro, which generates power using an emulsion, exhibited a pronounced decline in power generation during the latter stages of the experiment. In conclusion, the subsequent section of this paper will present a comprehensive analysis of the data obtained from the abovementioned series of experimental processes.

## 3. Results and Discussion

### 3.1. Single-Phase Liquid Energy Production

**Figure 4:**
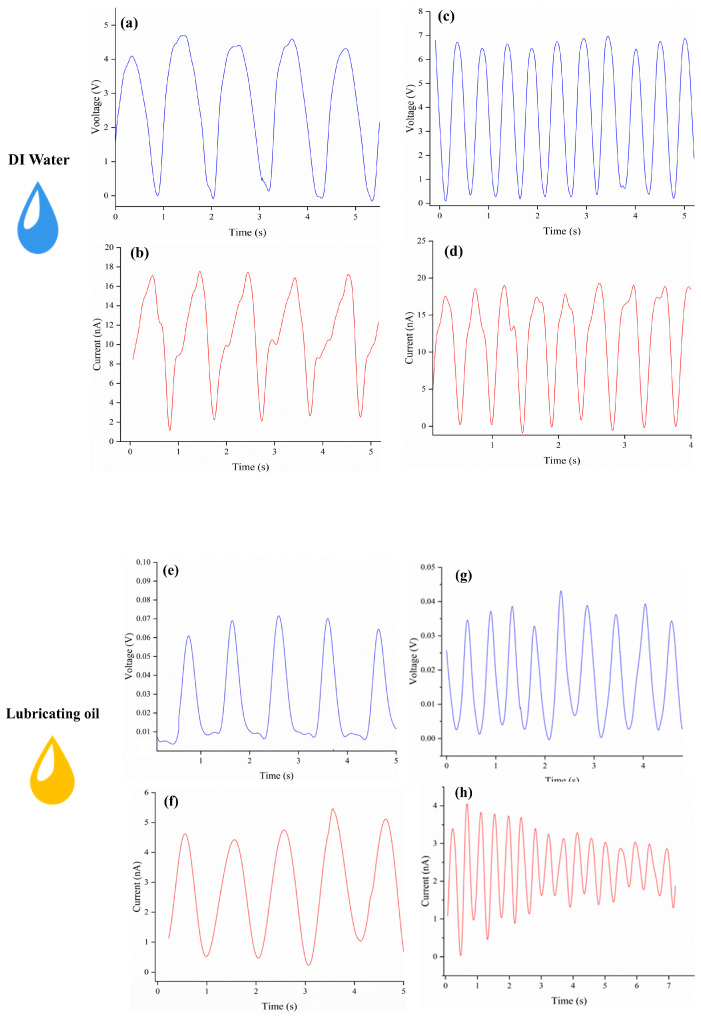

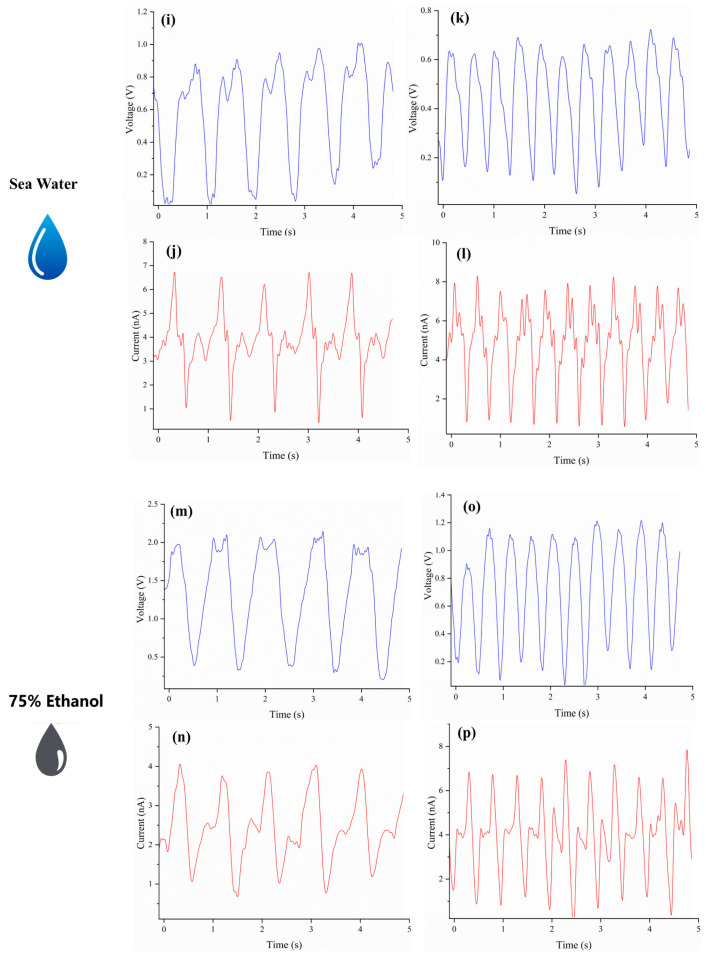
The power generation using a single-phase liquid triboelectric material. (**a**,**b**) Illustrate the generation of electricity by DI water in low-frequency tribological processes; (**c**,**d**) depict the same phenomenon in DI water of high-frequency tribological processes. (**e**,**f**) Depict the generation of electricity by lubricant oil in the low-frequency tribological processes. (**g**,**h**) Depict the lubricant oil in the high-frequency tribological processes. (**i**,**j**) Illustrate the generation of electricity by sea water in low-frequency tribological processes; (**k**,**l**) depict the same phenomenon in sea water of high-frequency tribological processes. (**m**,**n**) Illustrate the generation of electricity by 75% ethanol in low-frequency tribological processes; (**o**,**p**) depict the same phenomenon in 75% ethanol of high-frequency tribological processes.

In order to verify the effectiveness of the ML-TENG Pro power generation described in this paper, Figure 4 illustrates the power generation using a single-phase liquid triboelectric material. The experiments were conducted at two frequencies, namely 1 ± 0.5 Hz and 2 ± 0.5 Hz, with the former corresponding to a low-frequency and the latter representing a higher frequency. As illustrated in Figure 4a,b, the application of low-frequency triboelectric forces to DI water results in the generation of a voltage of approximately 4 V and a current of approximately 12 nA. As illustrated in Figure 4c,d, the voltage output of DI water during high-frequency triboelectric applications is approximately 6 V, while the current output is approximately 16 nA. The images of output voltage and current demonstrate a relatively stable pattern, although the output energy is relatively low. This discrepancy can be attributed to the compact size of the ML-TENG Pro, its PTFE membrane, and the absence of an adhesion coating between the PTFE and the pure copper membrane, resulting in a significant distance, l, between the main and reference electrodes, as indicated in Equation (3). Similarly, the power generation performance of the engine oil lubricant was evaluated using the abovementioned methodology. As illustrated in Figure 4e–h, the power generation effect of ML-TENG Pro in oil lubrication is minimal, with an output voltage of 0.03 to 0.06 V and an output current of 1 to 4 nA. It is hypothesized that this phenomenon may be caused by the absence of any oil-repellent properties exhibited by PTFE in relation to oil lubricants. The presence of a considerable quantity of adherent oil on the PTFE surface has the potential to impede the power generation effects observed in the friction process.

Furthermore, in this study, seawater and 75% ethanol solution were evaluated for their power generation performance in a similar manner. As illustrated in Figure 4i–l, ML-TENG Pro’s power generation in seawater is also at a low level, with an output voltage of 0.2 to 0.8 V and an output current of 1 to 7 nA. As mentioned in references [33,43] using seawater as a liquid power generation material, the power generation performance is not excellent. It is supposed that the electrolyte composition in seawater is more complex than that in deionized water, thus affecting the electron transfer and ion transfer processes. As illustrated in Figure 4m–p, the power generation of the ML-TENG Pro in a 75% ethanol solution is positioned between that of an oil-based organic solvent and an inorganic solvent, such as DI water, with an output voltage of 0.5 to 1.2 V and an output current of 2 to 8 nA. We suspected that the reason for this is that the polarity of the solution is weakened when ethanol and water are mixed, probably because the alkyl portion of the ethanol molecule weakens the dipole moment of the hydroxide bond.

### 3.2. Numerical Simulation of Multiphase Fluid Formation and “Air-Oil-Water” Three-Phase Fluid Power Generation

**Figure 5:**
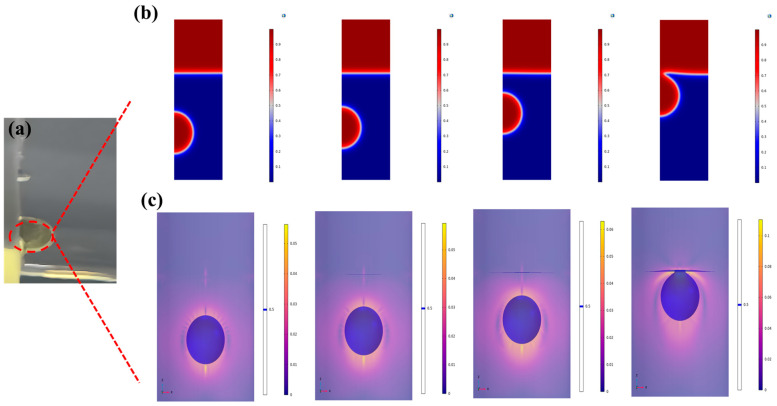
The simulation of the rising process of a drop of pure oil carried into DI water by ML-TENGPro. (**a**) Is a real shot of the oil drop, (**b**) is the process of the oil drop rising through the 2D numerical simulation, and (**c**) is the process of the oil drop rising through the 3D numerical simulation.

**Figure 6:**
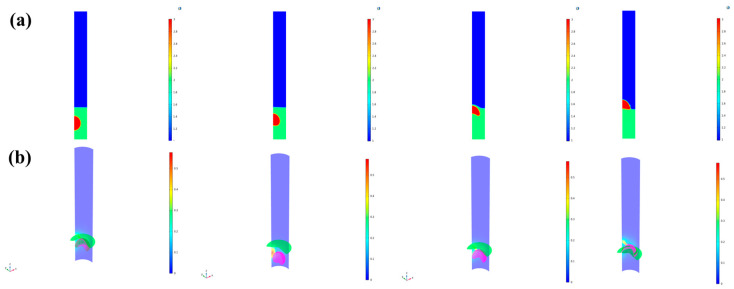
Bubbles carry the water phase across the oil–water interface or remain at the interface. (**a**) Is a 2D schematic diagram. (**b**) Is a 3D schematic diagram.

**Figure 7:**
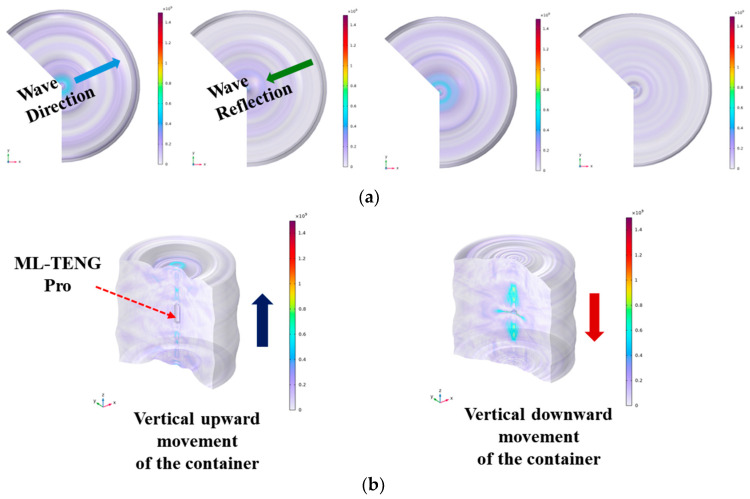
The ML-TENG Pro-induced transverse wave. (**a**) Is the top-down angle, and (**b**) is the transparent side view angle.

In the subsequent investigation, the utilization of an oil–water two-phase liquid material to validate the power generation performance of ML-TENG Pro presents a particularly fascinating and noteworthy process, as detailed in this paper. The general process can be described as follows: As the ML-TENG Pro device continuously reciprocates in the initial two-phase liquid material, a significant quantity of air is drawn into the original two-phase liquid, resulting in the formation of a gas–oil–water three-phase liquid. The Figure 5, Figure 6 and Figure 7, produced using COMSOL Multiphysics 6.0 software, illustrate the fluid phase change mechanism, from the stratification of the oil–water two-phase to the formation of an air–oil–water three-phase mixture. Furthermore, the generation of transverse waves due to the forced motion of the aforementioned multiphase mixture is described; this process assists the mechanism of material transport and diffusion within the multiphase liquid. In addition, this paper identifies three stages of phase change in the three multiphase triboelectric nanogenerator fluids, and records and analyzes the generated energy for each of these three phases. The quality of the mesh generated using COMSOL Multiphysics 6.0 software determines how accurate the numerical simulation is. The meshes generated in this paper are all around 0.93, which is enough to meet the software’s requirement for mesh accuracy.

As demonstrated in Figure 5, this section employed the “layer-flow, two-phase flow, and horizontal collection” numerical analysis method. The top and bottom surfaces utilize no-slip conditions, and the instantaneous solution tool is utilized. The right side of the liquid’s boundary is employed with the “wall” condition, and the free triangular mesh contains 10,372 elements. The smallest mass recorded was 0.6548, while the average mass was 0.9325.

As shown in Figure 6, the methodology for the generation of a three-phase gas–water–oil field can be delineated as follows: as the ML-TENG Pro introduces air into the lowest point of the initial oil–water two-phase liquid, the expelled gas ascends within the two-phase liquid environment. In accordance with the formula interpretation of F. Boyer et al. [49] on the hydrodynamics of three-phase fluids, the numerical analysis of phase-field-laminar three-phase flow is utilized, employing a transient solver. Additionally, the presence of wetted-wall conditions at the gas, water, and oil–liquid phase boundaries is considered. The present study delineates the water–oil-phase interfacial surface tension, denoted as $\sigma_{23}$, by invoking the computational method outlined in the literature. The adopted mesh configuration comprises a free triangular mesh, incorporating 36,153 elements. The minimum mass recorded is 0.645, while the average mass is 0.9523.

(7) $$V>V^{C}=\sqrt[{3/2}]{\frac{2\pi {\left(\frac{3}{4\pi }\right)}^{1/3}\sigma_{23}}{(\rho_{3}-\rho_{1})g}}$$

where the Surface Tension Coefficient between oil and water $\sigma_{23}$ is assumed to be 0.18; the densities of oil and air ($\rho_{3}$ = 0.86 and $\rho_{1}$ = 1.19) are also considered.

In this study, at a temperature of 20 °C, bubbles with a diameter exceeding 2 mm are observed to penetrate and flow into the oil phase. Bubbles with a diameter of 1 to 2 mm are retained at the oil–water interface. The entrapped aqueous phase within these bubbles, generated during ascent, gives them an A/O/W multiphase droplet structure from the inside out. The formation of a dense layer at the oil–water interface initially obstructs the upward movement of the bubble, which has a large volume.

Figure 7 illustrates the phenomenon of transverse waves generated by the vertical up-and-down movement of the ML-TENG Pro in a multiphase liquid. In this context, the ML-TENG Pro is regarded as the source of vibration, with the resulting transverse waves reflected upon contact with the container wall. The observed fluctuations facilitate the transport of A/O/W multiphase droplets, resulting in the rapid transition of liquids with pronounced stratification into a semi-emulsified state with less-distinct stratification.

In this section, the numerical analysis method of “single-phase flow—turbulence” is employed, and the conditions of no-slip and the use of a transient solver are continued. In this paper, the frequency of vertical displacement of ML-TENG is defined according to the experimental conditions, and the free triangular mesh “grid” is used, which contains 12,855 elements. The minimum mass is determined to be 0.6861, while the average mass is calculated to be 0.9526.

**Figure 8:**
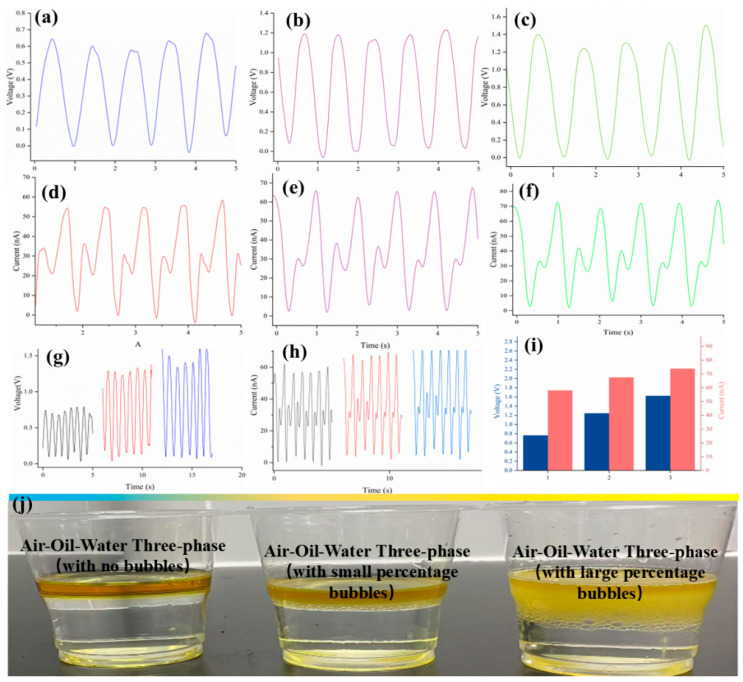
The power generation of ML-TENG Pro effect from the obvious oil–water interface to the fuzzy semi-emulsified interface. The image (**j**) is a genuine photograph of the three stages; (**a**–**c**) represent the voltage diagram; (**d**–**f**) are current graphs; (**g**–**i**) are the Vpp and nApp comparison charts for the three phases.

Figure 8 records the power generation effect from the obvious oil–water interface to the fuzzy semi-emulsified interface, while Figure 8i presents a real photo of the three stages of multiphase liquids classified within the context of this paper. As illustrated in Figure 8a,d during the phase of pronounced oil–water stratification, the power generated by the oil phase can be deemed insignificant in comparison, leaving only the power generation exhibited by the DI water. The subsequent situation is illustrated in Figure 8b,e, which depict the generation of a small number of A/O/W bubbles at the interface, accompanied by an increase in power generation. As illustrated in Figure 8c,f, a considerable number of A/O/W bubbles obscure the interface layer, resulting in the formation of a semi-emulsified liquid phase. This is accompanied by a notable increase in power generation.

Figure 8g,h depict the voltage and current outputs of the aforementioned stages. Figure 8i presents a comparative analysis of the performance status of the three stages of power generation. Figure 8g,h depict the voltage and current outputs of the aforementioned stages. Figure 8i presents a comparative analysis of the performance status of the three stages of power generation.

### 3.3. Discussion of the Emulsion Power Generation Phenomenon and Limiting Factors

As evidenced in the above study, this paper’s findings indicate that the power generation of the ML-TENG Pro exhibited a gradual increase, in conjunction with the formation of the semi-emulsion. This phenomenon may be attributed to the presence of air bubbles in the A/O/W liquid phase, which are encased in a water film on the exterior. Additionally, the microscopic oil–water interface exhibits a net electrical charge. Upon contact with these multiphase air bubbles, the PTFE layer of the ML-TENG Pro effectively interacts with them, leading to the conclusion that the bubbles can be regarded as “water droplets with enhanced electrical charge”.

**Figure 9:**
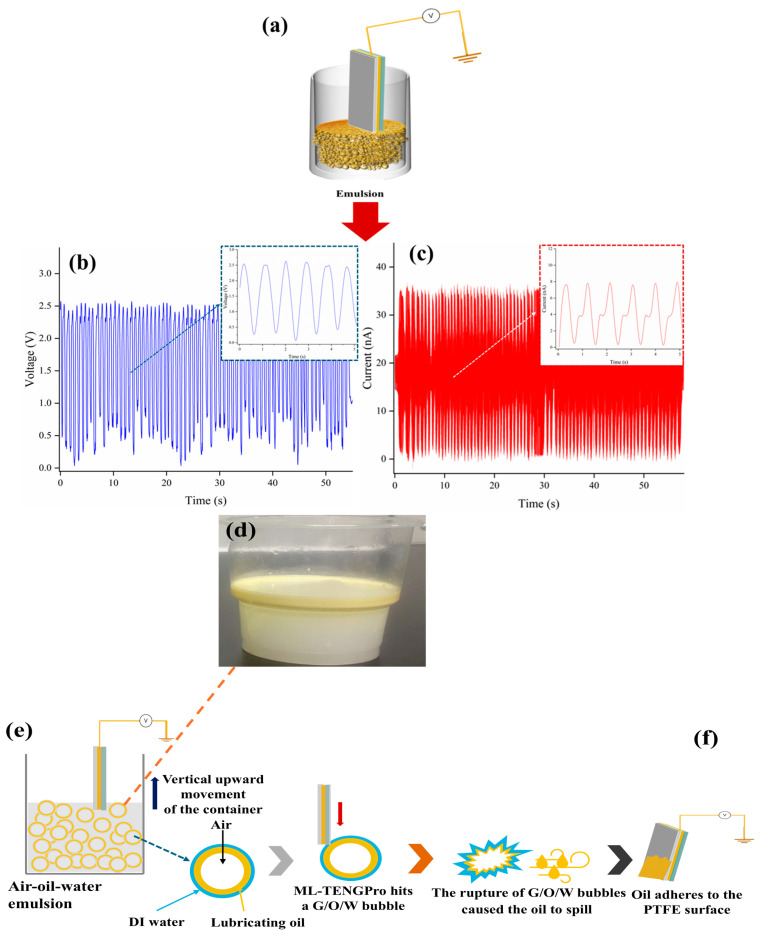
Experiment on the power generation performance of fully emulsified liquid. (**a**) describes how ML-TENG Pro enters the emulsion. voltage and current diagram (**b**,**c**). The figure (**e**,**f**) demonstrate the rationale behind the constrained power generation capacity of ML-TENG Pro in a fully emulsified liquid. (**d**) photograph of emulsion used in experiment.

In light of these considerations, a more ambitious approach is put forth in this paper. In this paper, an oil–water mixture solution with a volume ratio of 3:7 is used, and this solution is continuously stirred at high speed with a stirring rod, with the objective of obtaining a fully emulsified liquid, as demonstrated in Figure 9d. This emulsified liquid serves as the liquid phase dielectric material utilized in the development of triboelectric nanogenerators, as illustrated in Figure 9a. It is encouraging to observe that the fully emulsified A/O/W liquid, as illustrated in Figure 9b,c, displays a notable power generation capacity. This outcome corroborates the hypothesis of “charge storage enhanced droplets”, which was proposed in this paper. However, it is unfortunate that as the ML-TENG Pro reciprocating motion power generation experiment progresses, the power generation performance of the fully emulsified liquid gradually declines until it becomes undetectable.

As demonstrated by Figure 9e,f, the observed deterioration in the power generation performance is attributed to a process whereby the ML-TENG Pro reciprocating motion repeatedly disrupts the A/O/W emulsion, causing oil droplets to leak out and adhere to the PTFE layer of the ML-TENG Pro. The persistence of this phenomenon results in the accumulation of an oil film on the surface of the ML-TENG Pro, which ultimately impedes the generation of electricity.

## 4. Conclusions and Outlook

This paper proposes ML-TENG Pro as an improved sensor compared to previous single-electrode SL-TENG technology. ML-TENG Pro uses different liquid materials to generate electricity. In order to ascertain the stability of the energy output of ML-TENG Pro, the device was tested with DI water and oil lubricants, which exhibited different contact-based friction frequencies. The experiments presented in this paper also investigated the power generation performance of ML-TENG Pro in a two-phase liquid, air–oil–water three-phase liquids, and fully emulsified liquid.

The generation of the air–oil–water three-phase liquid in the experiment was analyzed using COMSOL Multiphysics 6.0 software, which demonstrated that the ML-TENG Pro carries a large amount of air into the original oil. During the vertical movement of the contact liquid phase, the water phase evidently underwent stratification, with the air mass rising in the mixed liquid and carrying part of the water phase. This resulted in the formation of a semi-emulsified layer at the oil–water interface. Subsequently, the power generation performance of the air–oil–water three-phase liquid is also described in this paper. Furthermore, an innovative approach was taken with a completely emulsified liquid as a power generation liquid, which also demonstrated a relatively satisfactory power generation performance. Based on the experimental results previously described in this paper, the following conclusions are presented:

(1) The power output of the ML-TENG Pro was found to be correlated with the frequency of contact with the liquid when a single liquid phase (e.g., deionized water, seawater) was used as the liquid power generation material. The size and thickness of the dielectric layer also affected the final power generation of the ML-TENG Pro.
(2) This paper proposed an air–oil–water (A/O/W) droplet structure model in the context of using multiphase liquids, such as air–oil–water, as liquid power generation materials. The model proposed that the A/O/W droplet is a “charge-storage-enhanced droplet”.
(3) Experiments performed with ML-TENG Pro power generation provided substantial support for a hypothesized model of droplet structure, namely the (A/O/W) droplet model, which has been termed the “charge-storage-enhanced droplet”. This is based on emulsified liquid power generation experiments.
(4) The PTFE triboelectric material used in ML-TENG Pro did not have enough oil-repellent properties to maintain effective power generation in the presence of lubricating oil, which caused continuous degradation of power generation.

The underlying rationale behind the utilization of the prefix “prototype” in the context of ML-TENG is elucidated by the outcomes of the aforementioned experiments. The performance of the ML-TENG power generation apparatus is found to be unstable when employed for power generation in mixed liquids comprising oil phases.

Consequently, the subsequent research will concentrate on the following three primary aspects:

(1) enhancing the oil repellency of solid dielectric materials;
(2) analyzing the power generation effect in emulsions with varying oil-to-water volume ratios;
(3) investigating the fundamental mechanism of generating multiphase droplet structures under diverse gas, oil, and water volume ratios.

Lastly, the design and fabrication of relevant small-scale self-supplied energy-sensing devices will be undertaken to ensure the successful realization of their engineering application value.

## Figures and Tables

**Table 1:** Single-phase liquid medium S-L TENGs in recent years.

Liquid Material Option	Solid-Phase Material Option	Liquid Flow Regime	Voltage	Current	Ref.
Deionized water	PTFE	Drops	5.25 V	5.2 mA	[33]
Tap water	FEP	Wave	43.2 V	5.1 mA	[34]
Rain	PTFE	Drops	4.6 V	0.42 mA	[33]
0.6 M NaCl aqueous solution	PTFE	Drops	1.6 V	0.16 mA	[33]
Isopropanol,	FEP	Flow	5 V	-	[35]
Acetone	FEP	Flow	6 V	-	[35]
Dimethyl sulfoxide	FEP	Flow	39 V	-	[35]
Polyalphaolefin 6 oils	PTFE	Flow	0.33 V	-	[36]
Paraffin oils	PTFE	Flow	0.32 V	-	[36]
Rapeseed oils	PTFE	Flow	0.1 V	-	[36]

**Table 2:** A general summary of L-S TENGs’ applications, based on the Single-Electrode mode.

Application Scene	Energy Source	Operating Mode	Ref.
Ocean energy harvest	Seawater	Hybrid mode	[43]
	Liquid	Freestanding mode	[44]
Self-powered sensor	Water	Single-Electrode mode	[45]
	Water	Single-Electrode mode	[44]
	NaCl solution	Single-Electrode mode	[46]
	Water flow	Single-Electrode mode	[46]
	Water droplet	Single-Electrode mode	[46]

## Data Availability

The original contributions presented in this study are included in the article. Further inquiries can be directed to the corresponding author.

## References

[1] Fan F.-R., Tian Z.-Q., Wang Z.L. (2012). Flexible triboelectric generator. Nano Energy.

[2] Choi Y.S., Kim S., Kar-Narayan S. (2021). Materials-related strategies for highly efficient triboelectric energy generators. Adv. Energy Mater..

[3] Luo H., Du J., Yang P., Shi Y., Liu Z., Yang D., Zheng L., Chen X., Wang Z.L. (2023). Human–machine interaction via dual modes of voice and gesture enabled by triboelectric nanogenerator and machine learning. ACS Appl. Mater. Interfaces.

[4] Xu W., Liu S., Yang J., Meng Y., Liu S., Chen G., Jia L., Li X. (2022). Self-Powered Flexible Handwriting Input Panel with 1D Output Enabled by Convolutional Neural Network. Nano Energy.

[5] Cheng T., Shao J., Wang Z.L. (2023). Triboelectric nanogenerators. Nat. Rev. Methods Prim..

[6] Luo J., Gao W., Wang Z.L. (2021). The triboelectric nanogenerator as an innovative technology toward intelligent sports. Adv. Mater..

[7] Bukhari M.U., Khan A., Maqbool K.Q., Arshad A., Riaz K., Bermak A. (2022). Waste to energy: Facile, low-cost and environment-friendly triboelectric nanogenerators using recycled plastic and electronic wastes for self-powered portable electronics. Energy Rep..

[8] Cao X., Xiong Y., Sun J., Xie X., Sun Q., Wang Z.L. (2023). Multidiscipline Applications of Triboelectric Nanogenerators for the Intelligent Era of Internet of Things. Nano-Micro Lett..

[9] Chao S., Ouyang H., Jiang D., Fan Y., Li Z. (2021). Triboelectric nanogenerator based on degradable materials. EcoMat.

[10] Chaturvedi A.K., Badatya S., Pappu A., Srivastava A.K., Gupta M.K. (2023). Sustainable robust waste-recycled ocean waterresistant fly ash-carbon nanotube nanocomposite-based triboelectric nanogenerator. Sustain. Energy Fuels.

[11] Chau N.M., Le T.H., La T.T.H., Bui V.-T. (2023). Industrially compatible production of customizable honeycomb-patterned poly (vinyl chloride) using food-wrapping waste for power-boosting triboelectric nanogenerator and ocean wave energy harvester. J. Sci. Adv. Mater. Devices.

[12] Chen A., Zhang C., Zhu G., Wang Z.L. (2020). Polymer materials for high-performance triboelectric nanogenerators. Adv. Sci..

[13] Cho S., Cha K., Kim B., Lee J., Park K., Chung S.-H., Song M., Heo D., Son J.-H., Choi M. (2023). Sustainable utilization of aging-deteriorated microplastics as triboelectric nanogenerator. Chem. Eng. J..

[14] Lin Z., Cheng G., Lin L., Lee S., Wang Z.L. (2013). Water-solid surface contact electrification and its use for harvesting liquid-wave energy. Angew. Chem. Int. Ed. Engl..

[15] Tang W., Chen B.D., Wang Z.L. (2019). Recent Progress in Power Generation from Water/Liquid Droplet Interaction with Solid Surfaces. Adv. Funct. Mater..

[16] Li X., Yeh M.-H., Lin Z.-H., Guo H., Yang P.-K., Wang J., Wang S., Yu R., Zhang T., Wang Z.L. (2015). Self-Powered Triboelectric Nanosensor for Microfluidics and Cavity-Confined Solution Chemistry. ACS Nano.

[17] Chen J., Guo H., Zheng J., Huang Y., Liu G., Hu C., Wang Z.L. (2016). Self-powered triboelectric micro liquid/gas flow sensor for microfluidics. ACS Nano.

[18] Vo C.P., Shahriar M., Le C.D., Ahn K.K. (2019). Mechanically active transducing element based on solid–liquid triboelectric nanogenerator for self-powered sensing. Int. J. Precis. Eng. Manuf.-Green Technol..

[19] Jeon S., Seol M., Kim D., Park S., Choi Y. (2016). Self-Powered Ion Concentration Sensor with Triboelectricity from Liquid–Solid Contact Electrification. Adv. Electron. Mater..

[20] Wu H., Chen Z., Xu G., Xu J., Wang Z., Zi Y. (2020). Fully Biodegradable Water Droplet Energy Harvester Based on Leaves of Living Plants. ACS Appl. Mater. Interfaces.

[21] Shi Q., Wang H., Wang T., Lee C. (2016). Self-powered liquid triboelectric microfluidic sensor for pressure sensing and finger motion monitoring applications. Nano Energy.

[22] Pal A., Chatterjee S., Saha S., Barman S.R., Choi D., Lee S., Lin Z.-H. (2020). A highly sensitive mercury ion sensor based on solid-liquid contact electrification. ECS J. Solid State Sci. Technol..

[23] Choi D., Tsao Y.-H., Chiu C.-M., Yoo D., Lin Z.-H., Kim D.S. (2017). A smart pipet tip: Triboelectricity and thermoelectricity assisted in situ evaluation of electrolyte concentration. Nano Energy.

[24] Lin S.-Q., Shao T.-M. (2017). Bipolar charge transfer induced by water: Experimental and first-principles studies. Phys. Chem. Chem. Phys..

[25] Lin S., Chen X., Wang Z.L. (2021). Contact electrification at the liquid–solid interface. Chem. Rev..

[26] Wang Z.L., Wang A.C. (2019). On the origin of contact-electrification. Mater. Today.

[27] Li S., Zhou Y., Zi Y., Zhang G., Wang Z.L. (2016). Excluding contact electrification in surface potential measurement using kelvin probe force microscopy. ACS Nano.

[28] Zhou Y.S., Liu Y., Zhu G., Lin Z.-H., Pan C., Jing Q., Wang Z.L. (2013). In situ quantitative study of nanoscale triboelectrification and patterning. Nano Lett..

[29] Lin S., Xu L., Zhu L., Chen X., Wang Z.L. (2019). Electron Transfer in Nanoscale Contact Electrification: Photon Excitation Effect. Adv. Mater..

[30] Xu C., Zi Y., Wang A.C., Zou H., Dai Y., He X., Wang P., Wang Y., Feng P., Li D. (2018). On the electron-transfer mechanism in the contact-electrification effect. Adv. Mater..

[31] Xiang T., Chen X., Sun H., Liu D., Jiang Y., Chen S., Xie Y., Zhang S. (2024). Advances in liquid-solid triboelectric nanogenerators and its applications. J. Mater. Sci. Technol..

[32] Jin X., Yang W., Gao X., Li Z. (2020). Analysis and Modeling of the Complex Dielectric Constant of Bound Water with Application in Soil Microwave Remote Sensing. Remote Sens..

[33] Cai C., Luo B., Liu Y., Fu Q., Liu T., Wang S., Nie S. (2022). Advanced triboelectric materials for liquid energy harvesting and emerging application. Mater. Today.

[34] Cao X., Zhou H., Zhou Y., Hu Y., Wang Y., Wang Z.L., Sun Q. (2023). High Performance Rotary-Structured Triboelectric-Electromagnetic Hybrid Nanogenerator for Ocean Wind Energy Harvesting. Adv. Mater. Technol..

[35] Zhao J., Wang D., Zhang F., Liu Y., Chen B., Wang Z.L., Pan J., Larsson R., Shi Y. (2021). Real-Time and Online Lubricating Oil Condition Monitoring Enabled by Triboelectric Nanogenerator. ACS Nano.

[36] Hu S., Shi Z., Zheng R., Ye W., Gao X., Zhao W., Yang G. (2020). Superhydrophobic liquid–solid contact triboelectric nanogenerator as a droplet sensor for biomedical applications. ACS Appl. Mater. Interfaces.

[37] Xu S., Feng Y., Liu Y., Wu Z., Zhang Z., Feng M., Zhang S., Sun G., Wang D. (2021). Gas-solid two-phase flow-driven triboelectric nanogenerator for wind-sand energy harvesting and self-powered monitoring sensor. Nano Energy.

[38] Yang L., Wang Y., Zhao Z., Guo Y., Chen S., Zhang W., Guo X. (2020). Particle-laden droplet-driven triboelectric nanogenerator for real-time sediment monitoring using a deep learning method. ACS Appl. Mater. Interfaces.

[39] Huang T., Sun W., Liao L., Zhang K., Lu M., Jiang L., Chen S., Qin A. (2023). Detection of Microplastics Based on a Liquid-Solid Triboelectric Nanogenerator and a Deep Learning Method. ACS Appl. Mater. Interfaces.

[40] Dong Y., Xu S., Zhang C., Zhang L., Wang D., Xie Y., Luo N., Feng Y., Wang N., Feng M. (2022). Gas-liquid two-phase flow-based triboelectric nanogenerator with ultrahigh output power. Sci. Adv..

[41] Jiang P., Zhang L., Guo H., Chen C., Wu C., Zhang S., Wang Z.L. (2019). Signal Output of Triboelectric Nanogenerator at Oil–Water–Solid Multiphase Interfaces and its Application for Dual-Signal Chemical Sensing. Adv. Mater..

[42] Bai Y., Feng H., Li Z. (2022). Theory and applications of high-voltage triboelectric nanogenerators. Cell Rep. Phys. Sci..

[43] Wu M., Guo W., Dong S., Liu A., Cao Y., Xu Z., Lin C., Zhang J. (2022). A hybrid triboelectric nanogenerator for enhancing corrosion prevention of metal in marine environment. npj Mater. Degrad..

[44] Wei X., Zhao Z., Zhang C., Yuan W., Wu Z., Wang J., Wang Z.L. (2021). All-weather droplet-based triboelectric nanogenerator for wave energy harvesting. ACS Nano.

[45] Liu G., Xiao L., Chen C., Liu W., Pu X., Wu Z., Hu C., Wang Z.L. (2020). Power cables for triboelectric nanogenerator networks for large-scale blue energy harvesting. Nano Energy.

[46] Rui P., Zhang W., Zhong Y., Wei X., Guo Y., Shi S., Liao Y., Cheng J., Wang P. (2020). High-performance cylindrical pendulum shaped triboelectric nanogenerators driven by water wave energy for full-automatic and self-powered wireless hydrological monitoring system. Nano Energy.

[47] Suh I.-Y., Jeon J., Park M.J., Ryu H., Park Y.J., Kim S.-W. (2024). Recent Studies on Solid–Liquid Contact Electrification. ACS Appl. Electron. Mater..

[48] Wu H., Li B., Zhou F. (1999). Experimental study of the flow pattern of oil-gas-water three-phase flow in a horizontal pipe. Oil Gas Storage Transp..

[49] Boyer F., Lapuerta C., Minjeaud S., Piar B., Quintard M. (2009). Cahn–Hilliard/Navier–Stokes Model for the Simulation of Three-Phase Flows. Transp. Porous Media.

